# A viral journey to the brain: Current considerations and future developments

**DOI:** 10.1371/journal.ppat.1008434

**Published:** 2020-05-21

**Authors:** Nilda Vanesa Ayala-Nunez, Raphael Gaudin

**Affiliations:** 1 Institut de Recherche en Infectiologie de Montpellier (IRIM)—CNRS, Montpellier, France; 2 Université de Montpellier, Montpellier, France; Mount Sinai School of Medicine, UNITED STATES

Viral infections of the central nervous system (CNS) represent a significant burden to human health worldwide. Neurotropic viruses must travel from a body entry gate up to the CNS, where they infect local cells and potentially cause neurological disorders. The brain is protected from blood-borne pathogens by the so-called blood–brain barrier (BBB), an endothelial cell wall exhibiting extremely low permeability. Bacteria, fungi, parasites, and viruses have evolved various powerful strategies to reach the brain [1–3]. However, it is unclear whether the different strategies coexist or a main pathway prevails while the others are marginal. Here, we describe the main strategies for viruses to cross the BBB, with a particular focus on flaviviruses, which represent important emerging human neurotropic pathogens. The aim of this review is to put into perspective the four main ways to cross the BBB and to re-visit these concepts in the light of new technical developments.

## Question 1. What are the different paths viruses can use to cross a tight endothelial cell wall?

**Fig 1:**
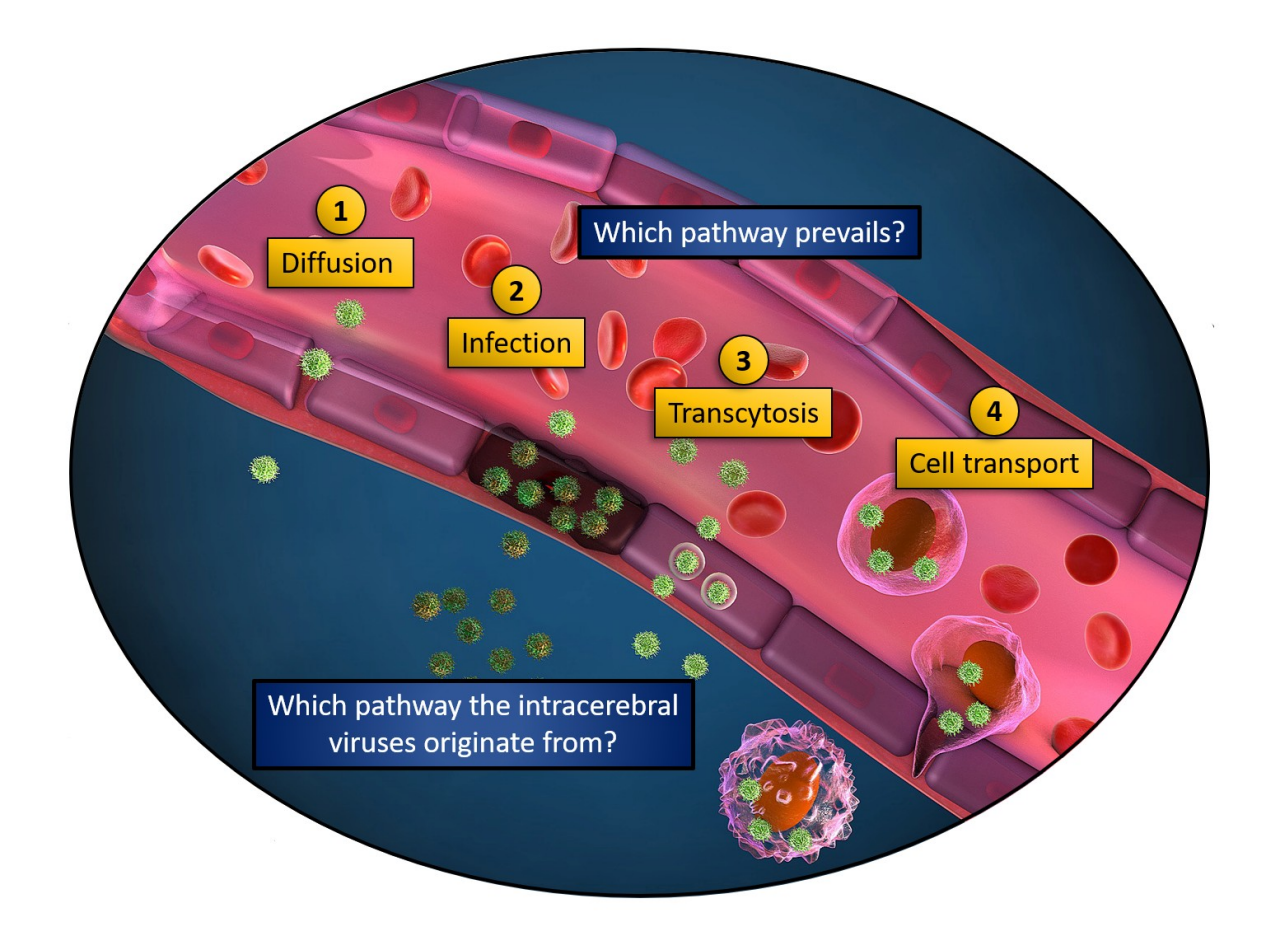
Ways viruses can use to cross the BBB. Illustration of a blood vessel and the four nonexclusive ways viruses may employ to reach adjacent tissues: 1. Diffusion (passive-aggressive way): Viruses freely diffuse when endothelium integrity is altered. 2. Infection (energetic way): The endothelium is infected and viruses released on the other side. 3. Transcytosis (commuting way): The circulating virions are endocytosed by endothelial cells and exocytosed on the other side, in the absence of productive infection. 4. Cell transport (Trojan horse way): Leukocytes are carrying the circulating viruses through the endothelial cell wall. BBB, blood–-brain barrier.

The four main ways described in the literature and represented in Fig 1 are:

1. Passive diffusion (the passive aggressive way): In this model, viruses passively diffuse in-between endothelial cells. This is possible in loose or injured endothelia, or upon induced permeabilization.
2. Endothelial cell infection (the energetic way): In this model, viral tropism is compatible to endothelial cell infection. Virus replication in endothelial cells allows virus release on the basolateral membrane of the endothelium, therefore releasing infectious viral particles toward the adjacent tissue.
3. Virus transcytosis (the commuting way): In this model, endothelial cells are not infected but still uptake circulating viral particles into nondegradative endosomal vesicles that are then released on the other side of the endothelial cell wall.
4. Cell-associated virus transport (the Trojan horse way): In this model, viruses infect or are carried by blood circulating cells, which undergo blood-to-tissue transmigration throughout the endothelial cells (via paracellular or transcellular migration).

## Question 2. What is the interplay between the various pathways?

Numerous primary research studies have investigated viral dissemination to the CNS, but none of them compared in parallel the different ways a virus can use to cross the BBB. Since proving the existence of one way does not disproof the existence of the others, one can speculate that they actually coexist and may be influenced and triggered by each other.

For instance, passive diffusion (way 1) may be tightly connected to productive infection of endothelial cells (way 2), as virus-induced cell death can result in BBB leakage. Flaviviruses have been reported to infect endothelial cells, at least in vitro. However, the picture is less clear in clinical samples and mouse models (for detailed reviews, see [1, 4]). For instance, no endothelial cell infection was found in clinical samples of a Zika virus (ZIKV)-infected brain from a fetus with severe microcephaly [5], and thus, ZIKV crossing to the brain is likely independent of a productive BBB infection.

Way 1 involves passive diffusion of viral particles through the BBB, but addressing it as “passive” is misleading. Indeed, for diffusion to happen, earlier virus-induced perturbations should have occurred, which in turn caused the BBB to be leaky. This apparently “passive” diffusion is rather a passive-aggressive strategy. A leaky endothelium can be the result of either (A) the direct infection of endothelial cells (way 2) that compromises endothelial impermeability, (B) the induction of a strong inflammatory response—the so-called “cytokine storm”—or (C) the release of propermeable viral proteins into the blood stream. These nonexclusive options have all been proposed for the Flaviviridae family, and, in particular, the third scenario was recently mechanistically deciphered, occurring through nonstructural protein 1 (NS1)-mediated vascular leakage [6, 7].

Virus transcytosis (way 3) is difficult to experimentally discriminate from productive infection (way 2), as exemplified recently by Papa and colleagues. [8]. To date, no evidence supports the existence of this pathway for Flaviviridae BBB crossing. Interestingly, dengue virus (DENV) infection modulates transcytosis of soluble factors [9], but the viral particle itself was not shown to be readily transported through this route.

The Trojan horse strategy (way 4) is an attractive and conceptually relevant option that was proposed almost 40 years ago for the Visna virus [10]. It extends beyond virology, as bacteria, parasites and synthetic nanodelivery carriers are also employing this strategy [2, 11–13]. This pathway confers a significant advantage to the virus because the carrier cells hide it from immune surveillance. The Trojan horse can be a more efficient strategy to crossing the BBB than a cell-free virus. When comparing cell-associated versus cell-free ZIKV dissemination using cerebral organoids beneath an endothelial layer, it was shown that free virions were disseminating slower than monocyte-associated ZIKV [5].

In these scenarios, it is very difficult to definitely ensure that one way precludes all the others from happening. On the contrary, one way may instead trigger the other ones. Then, the question is not anymore whether they coexist but rather which way comes first and which way contributes the most to neuroinvasion.

## Question 3. Which way comes first? A chicken-and-egg situation

For the Japanese encephalitis virus (JEV) and West Nile virus (WNV), reports suggest that first, virions enter the CNS with an integer BBB (by an unknown way) and, in a second step, disrupt the BBB through inflammatory signals [14, 15]. In the case of DENV, the Harris lab reported that vascular permeability was not cytokine-dependent but rather mediated by the flavivirus NS1 protein [6, 16]. In this latter case, mouse injection of the NS1 protein from WNV, DENV, and ZIKV (but not JEV) showed increased brain vasculature leakage at three days postinjection. One scenario could be that DENV infects cells (different from endothelial cells), leading to NS1 release to the blood stream, therefore inducing vascular leakage and increased passive diffusion of the virus to the BBB. Yet, it remains unknown whether the kinetics of NS1-mediated vascular leakage precedes or follows inflammation-induced BBB permeability.

In the Trojan horse strategy, the question is rather to determine whether the immune infected cells found in the brain correspond to the first wave of invasion or to secondary infiltrations responding to an already established brain infection. On one hand, immune cells readily migrate to the brain upon inflammation cues (for review, see [17]), but on the other hand, the virus may need to first reach the brain to induce this neuro-inflammation. These two options are both relevant but particularly challenging to discriminate in vivo. Although immune cells transmigrate vastly less in the absence of stimuli, it was previously observed by microscopic analyses of mouse brain tissue sections that a low (but nonnull) number of monocytes can patrol across the BBB [18]. Moreover, monocytes are efficient cellular Trojan horses for drug delivery to the brain in the absence of immunological signals [13]. Thus, it is reasonable to think that inflammation plays a significant role in viral BBB crossing and in the worsening of virus-induced neuropathology, but it may not be critical for initial cerebral colonization.

These observations highlight the importance of studying the temporality of events in greater details to map the sequential appearance of viral neuroinvasion, BBB leakage, and inflammation under infection conditions in relevant in vivo or ex vivo models.

## Question 4. What are the upcoming methodological approaches to study viral BBB crossing?

From an experimental point of view, studying all four pathways simultaneously is very challenging. In vivo, most of the flavivirus neuroinvasion studies have been performed in mice (see for instance [6, 8, 14, 15, 19]), focusing on a single way. In fixed samples, one could monitor several neuroinvasion pathways in parallel (for instance endothelial infection (way 2) and leukocyte infiltration (way 4)), but it would not discriminate whether it is the cause or the consequence of neuroinvasion. Spatiotemporal dynamics is an attractive approach to address such limitation, requiring 3D imaging of live infected mice. Although a few labs have achieved such challenging in vivo imaging in principle [20, 21], the low number of events, the thickness of the region to be imaged, and the relatively large time window of imaging are technical limitations for the use of the mouse model. In contrast, the transparency and the numerous tools available for imaging and genetic manipulation represent interesting advantages for the use of the zebrafish embryo in neuroinvasion studies. Recently, a zebrafish embryo model was developed to observe the transmigration through endothelia of ZIKV-infected human monocytes [5], a xenotypic transfer previously characterized [22]. The zebrafish model was also used to study neuroinvasion of two arboviruses, showing that chikungunya virus (CHIKV) was mainly using endothelial infection (way 2), while Sindbis virus (SINV) was mostly following peripheric axonal transport to reach the brain [23]. Although very attractive for spatiotemporal cell biology analyses, zebrafish infection does not recapitulate neuropathology observed in mammals, and thus, other complementary approaches are still needed.

Besides in vivo models, emerging in vitro systems of human endothelial barriers represent appealing strategies to gain further molecular insights onto viral neuroinvasion. To discriminate between the four ways presented in Fig 1, an ideal system should combine the following requirements: (A) measure endothelial permeability in real time, (B) have a reporter system to monitor productive infection, (C) track single viral particles, and (D) recapitulate bloodstream-like sheer stress. Moreover, the system should closely mimic an actual vasculature, including the possibility to coculture various cell types involved in BBB formation and maintenance and preserving the tube-shaped 3D architecture of the endothelial cells.

BBB spheroids made of human brain microvascular endothelial cells (HBMEC) were used to study ZIKV-induced THP-1 cells transmigration [24]. Recently, more physiological techniques taking advantage of human pluripotent stem cell (hPSC)-derived BBB organoids have been developed to obtain 3D human blood vessels with morphological, functional, and molecular features of human microvasculature [25]. The most evolved in vitro technique to date is probably the functional vasculature-like system grown within human cortical organoids derived from embryonic stem cells [26].

A common limitation of the organoid approach however is that they do not allow the application of a luminal flow. In that regard, original lithography-based microfluidic chips are being employed to combine vessel architecture and flow dynamics [27–29]. These innovative systems could provide quantitative temporal and spatial information to evaluate in parallel the contribution of each of the ways described in Fig 1. Although very attractive, these new methods may not be straightforward to implement in neophyte labs, but their rapid democratization should lead to exciting new discoveries in a near future.

## Conclusions

Common and divergent mechanisms have been evolved by the *Flavivirus* genus members to reach the CNS. We propose that these ways of crossing the BBB are not mutually exclusive but that they likely coexist and contribute to each other. However, a major difficulty faced by researchers includes the fact that the envisioned pathways (Fig 1), as well as unforeseen ones, are difficult to experimentally segregate and individually interrogate. The powerful in vitro BBB models recently developed could pave the way toward important breakthroughs in the coming years. We believe that engineering better in vitro cell walls will be key to spatiotemporally disentangle the mechanisms leading to flavivirus neuroinvasion.
